# SUMOylation and NEDDylation in Primary and Metastatic Cancers to Bone

**DOI:** 10.3389/fcell.2022.889002

**Published:** 2022-04-06

**Authors:** Marta Gomarasca, Giovanni Lombardi, Paola Maroni

**Affiliations:** ^1^ Laboratory of Experimental Biochemistry and Molecular Biology, IRCCS Istituto Ortopedico Galeazzi, Milano, Italy; ^2^ Department of Athletics, Strength and Conditioning, Poznań University of Physical Education, Poznań, Polska

**Keywords:** post-translational modifications (PMTs), ubiquitin, SUMO, NEDD8, bone metastasis, primary bone tumors

## Abstract

Post-translational modifications comprise series of enzymatically-driven chemical modifications, virtually involving the entire cell proteome, that affect the fate of a target protein and, in turn, cell activity. Different classes of modifications can be established ranging from phosphorylation, glycosylation, ubiquitination, acetylation, methylation, lipidation and their inverse reactions. Among these, SUMOylation and NEDDylation are ubiquitin-like multi-enzymatic processes that determine the bound of SUMOs and NEDD8 labels, respectively, on defined amino acidic residues of a specific protein and regulate protein function. As fate-determinants of several effectors and mediators, SUMOylation and NEDDylation play relevant roles in many aspects of tumor cell biology. Bone represents a preferential site of metastasis for solid tumors (e.g., breast and prostate cancers) and the primary site of primitive tumors (e.g., osteosarcoma, chondrosarcoma). Deregulation of SUMOylation and NEDDylation affects different aspects of neoplastic transformation and evolution such as epithelial-mesenchymal transition, adaptation to hypoxia, expression and action of tumor suppressors and oncogenic mediators, and drug resistance. Thereby, they represent potential therapeutic targets. This narrative review aims at describing the involvement and regulation of SUMOylation and NEDDylation in tumor biology, with a specific focus on primary and secondary bone tumors, and to summarize and highlight their potentiality in diagnostics and therapeutic strategies.

## 1 Post-Translational Modifications

Post-translational modifications (PTMs) play pivotal roles in several processes, such as protein stability and trafficking, binding affinity, signaling regulation, but also in the context of more general processes associated with cell fate, such as epithelial-mesenchymal transformation and apoptosis. The existence of different types of PTMs increases the functional diversity of the proteome, and, as a consequence, most of the aspects of normal cell biology and/or pathology are influenced. PTMs include phosphorylation, glycosylation, ubiquitination, SUMOylation, NEDDylation, acetylation, methylation, lipidation, and the relative reverse reactions; each of these modifications results from multistep processes, achieved and fine-tuned by the activity of enzymatic cascades. Deregulations of PTM steps are well documented in different types of cancer and cover a significant role in tumor pathogenesis (Chen et al., 2020; Xue et al., 2020). Consequently, targeting PTMs may represents a potential strategic approach in anticancer therapy.

Of particular interest are the PTMs with ubiquitin-like proteins (Ubls), a family of proteins that share similar structure and evolutionary relationship with ubiquitin (Ub), the first and best known member of this family. Ubls carry out important roles in cell fission, endocytic trafficking, as well as in higher function like immune response, neoplastic transformation. Two types of Ubls have been reported in humans: type I-Ubls, including SUMO1-5, NEDD8, ATG8, and ATG12, that share the enzymatic cascade that leads to the covalent binding of the Ubls to the protein target typical of the ubiquitin pathway, and type II-Ubls that do not act *via* any covalent binding (Cappadocia and Lima, 2018; Ilic et al., 2022).

The best-characterized Ubls are SUMO (small ubiquitin-like modifier) and NEDD8 (neural precursor cell-expressed developmentally downregulated 8) (Gatel et al., 2020).

In this review, we shortly describe the ubiquitin pathway, as it is the best described PTM mechanism and the basis for the other Ubls-mediated PTMs, and we explore in depth the role of the PTMs with SUMO, SUMOylation, and NEDD8, NEDDylation, focusing on primary bone cancers and metastatic cancer to bone.

### 1.1 Ubiquitination

**FIGURE 1:**
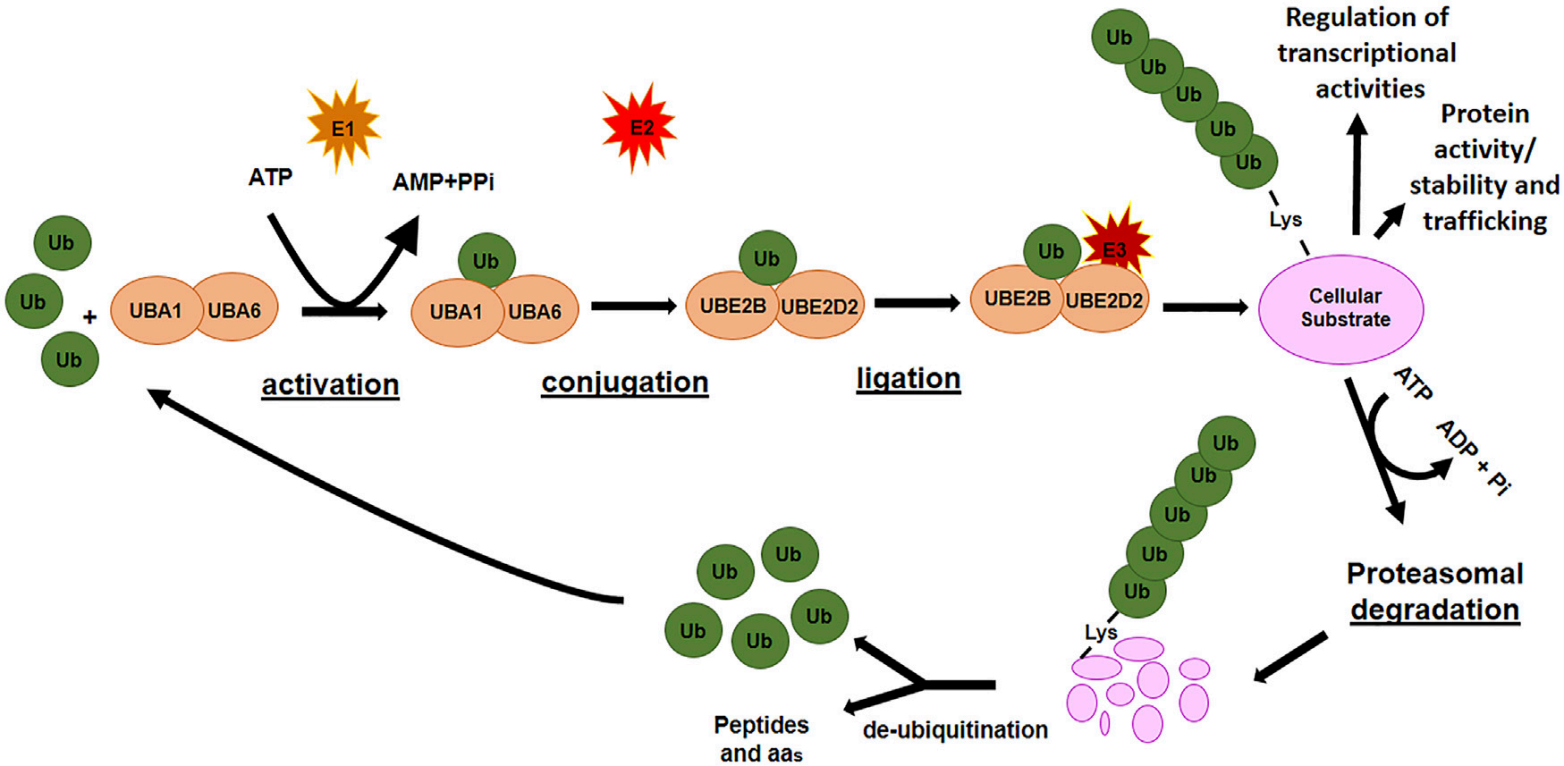
The ubiquitination pathway.

Ubiquitination is a multistep enzymatic cascade that leads to the attachment of ubiquitin (Ub) units (as either a single or poly Ub-chain, termed as mono- or poly-ubiquitination, respectively) to specific residues of a protein with a consequent control on protein abundance, function, and trafficking. The first step in ubiquitination is the activation of free-Ub by an E1 ubiquitin-activating enzyme in an ATP-dependent manner. The activated Ub is transferred to an E2 ubiquitin-conjugating enzyme and bound to a substrate protein through the actions of an E3 ubiquitin ligase enzyme, which identifies specific lysine residues on the target proteins (Figure 1). Finally, the ubiquitinated protein is usually recognized by the 26S proteasome and degraded into small peptides and amino acids. The ubiquitin-proteasome system ensures the removal of potentially harmful proteins as those damaged, unnecessary, or misfolded. However, different modes of ubiquitination lead to different substrate fates. Monoubiquitination is frequently related to the regulation of processes such as DNA repair and gene expression while multiubiquitination can regulate receptor endocytosis (Passmore and Barford, 2004). Polyubiquitination of Lys at different positions (i.e., K11 and K48) generally destines the protein to proteasomal degradation, whereas K63-linked poly-Ub chain can function in signal transduction, kinase activation, and endocytosis (Sadowski et al., 2012).

De-ubiquitinating enzymes (DUBs) remove poly-ubiquitin chains from proteins to maintain intracellular ubiquitin levels. The interplay between ubiquitinating and de-ubiquitinating enzymes is necessary to maintain cellular homeostasis. Since its involvement in numerous cellular processes, the failure to keep ubiquitination tightly controlled favors the development and progression of many pathologies, including cancers (Chen and Lu, 2015; Zou and Lin, 2021).

### 1.2 SUMOylation

SUMOylation (SUMO modification) is a dynamic and reversible PTM involved in different cellular processes: nuclear-cytoplasmic transfer, translational regulation, apoptosis, protein stability, genomic integrity, stress response, chromatin remodeling, and cell-cycle regulation (Melchior, 2000; Hay, 2005). It is the most studied Ubl-dependent PTM and it has been calculated that more than 6,000 proteins are targets for SUMOylation (Hendriks et al., 2017). SUMO proteins are members of a family of widely distributed Ubls found in all eukaryotic cells. They are proteins of ca. 10 kDa and share a similar three-dimensional structure with Ub even though they have only 20% homology in the amino acids sequence (Geiss-Friedlander and Melchior, 2007). SUMO proteins covalently bind cellular proteins to modify their functions, *via* different topologies: mono- multi- or poly-SUMOylation (Geiss-Friedlander and Melchior, 2007). Five subtypes of SUMO proteins are expressed in mammals: SUMO1-5. SUMO1-3 are ubiquitously expressed, with SUMO1 mainly targeting proteins in physiological states, and SUMO2/3 involved in stress responses. SUMO2 and SUMO3 have a specific N-terminus that allows the formation of multimers. SUMO4 and SUMO5 are the least well known SUMO paralogues, although their expression is restricted to specific tissues: SUMO4 is mainly expressed in kidney, spleen and lymph nodes, while SUMO5 is expressed only in few tissues, especially blood and testis (Bouchard et al., 2021; Yuan et al., 2021).

**FIGURE 2:**
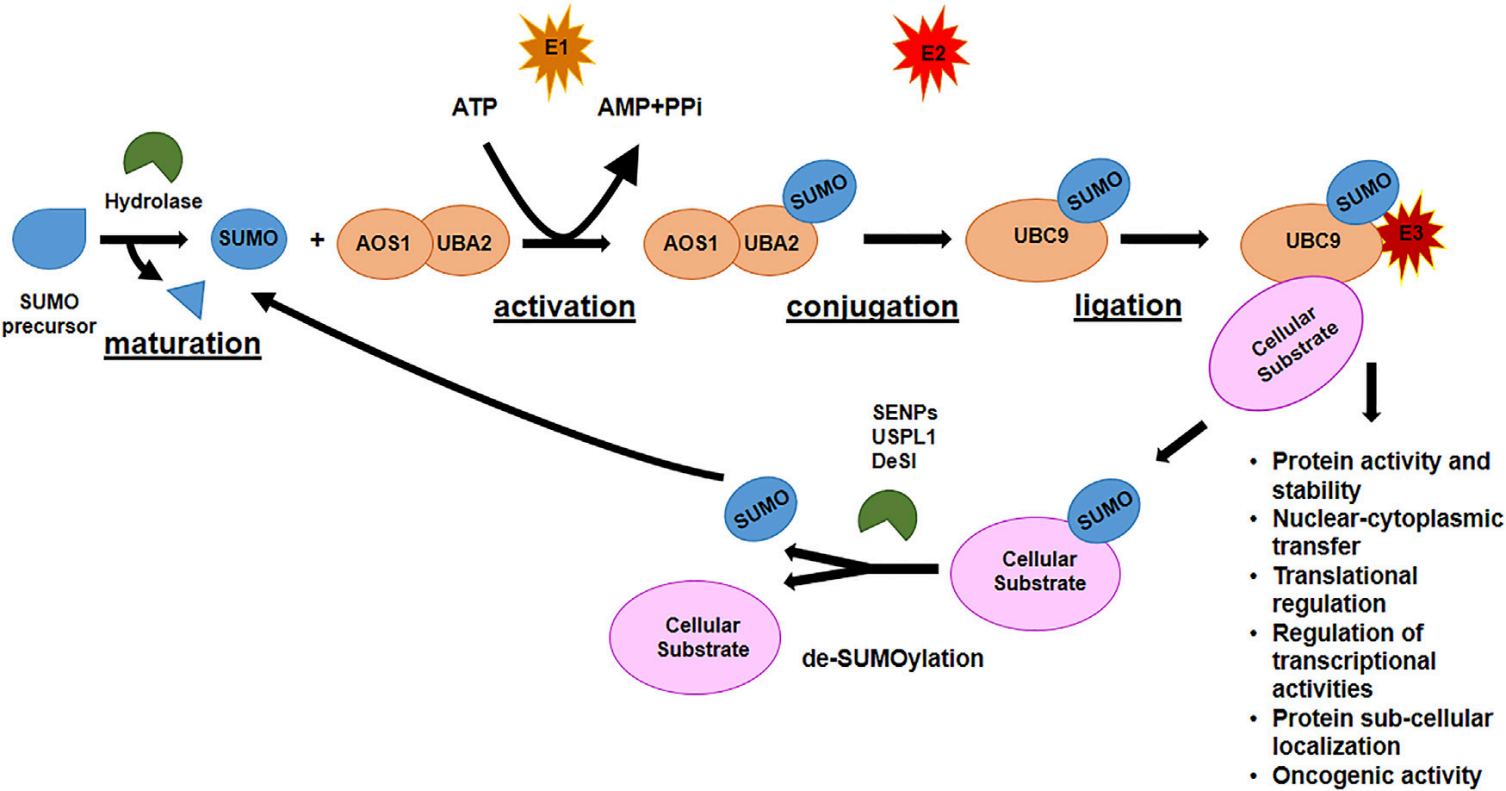
The SUMOylation pathway.

Like ubiquitination, SUMOylation is a multi-step process and involves a cascade of three classes of enzymes (Figure 2). After maturation of SUMO precursor, carried out by specific proteases, E1 activating enzyme, a heterodimer composed of two subunits, AOS1 and UBA2, activates the C-terminus of SUMO protein in an ATP-dependent reaction. SUMO is transferred to the E2 conjugating enzyme, Ubiquitin-conjugating enzyme 9 (Ubc9), the only E2 enzyme in SUMOylation process, at the Cys residue in the catalytic site of Ubc9 *via* a thioester bond. Finally, SUMO is transferred to the substrate through the formation of a thioester bridge between the Gly residue at the C-terminus of SUMO and the recipient Lys of the target protein. Different E3 ligase enzymes [Ran-binding protein 2 (Ranbp2), protein inhibitor of activated STAT (PIAS) family, NSE2 or MMS21, etc...] accelerate the rate of the final step of SUMOylation by promoting the covalent attachment of SUMO onto its substrates (Geiss-Friedlander and Melchior, 2007). As for ubiquitination, SUMOylation can be reverted by SUMO-specific proteases, of which the best known is the family of Sentrin/SUMO-specific proteases (SENP1-3 and SENP5-7), that, in mammalian cells, de-conjugates the molecules attached to the substrates (de-SUMOylation) and reverts the signaling. Each SENP has preferences for specific SUMO proteins: for example, SENP1-2 de-conjugate SUMO1 and SUMO2/3; SENP3-5 are specific for SUMO2/3; SENP6-7 de-conjugate poly-chains of SUMO2/3 (Tokarz and Wozniak, 2021). Due to their function, SENPs are pivotal regulators of the SUMOylation process and regulatory function. Other classes of SUMO proteases have been recently identified: DeSI-1 and DeSI-2, two isopeptidases with restricted target specificity as they were found to de-SUMOylate the transcription factor BZEL but not other SENPs substrate such as PML (Shin et al., 2012), and the highly specific SUMO isopeptidase ubiquitin-specific protease-like 1 (USPL1), with preference for de-SUMOylating SUMO2/3 (Schulz et al., 2012).

SUMOylation is characterized by the covalent and reversible isopeptide bond between the C-terminal Gly residue of SUMO and the Lys ε-amino group in the recipient protein. In particular it has been identified a specific SUMO consensus motif ΨKXE present in the substrate, in which *Ψ* represents a hydrophobic residue, K is the Lys acceptor residue, X may be any amino acid, and E is a glutamic acid residue (Gareau and Lima, 2010; Hendriks et al., 2014). This SUMOylation site is present in the majority of the targets; however, SUMO acceptor sites that do not contains the ΨKXE exist (Hietakangas et al., 2006; Yang et al., 2006).

The SUMOylation process is crucially involved in the tumorigenesis; indeed, dysregulation of SUMOylation enzymes, as well as abnormal SUMOylation of target proteins, has been found associated with multiple types of cancer.

SUMO can bind directly oncoproteins or indirectly oncosuppressors and, in both cases, by modifying their activity and/or their fate (e.g., degradation) it stimulates neoplastic growth. SUMOylation also intervenes in telomere length homeostasis and maintenance of genomic integrity and chromosome replication, processes related to carcinogenesis (Peuscher and Jacobs, 2012).

### 1.3 NEDDylation

**FIGURE 3:**
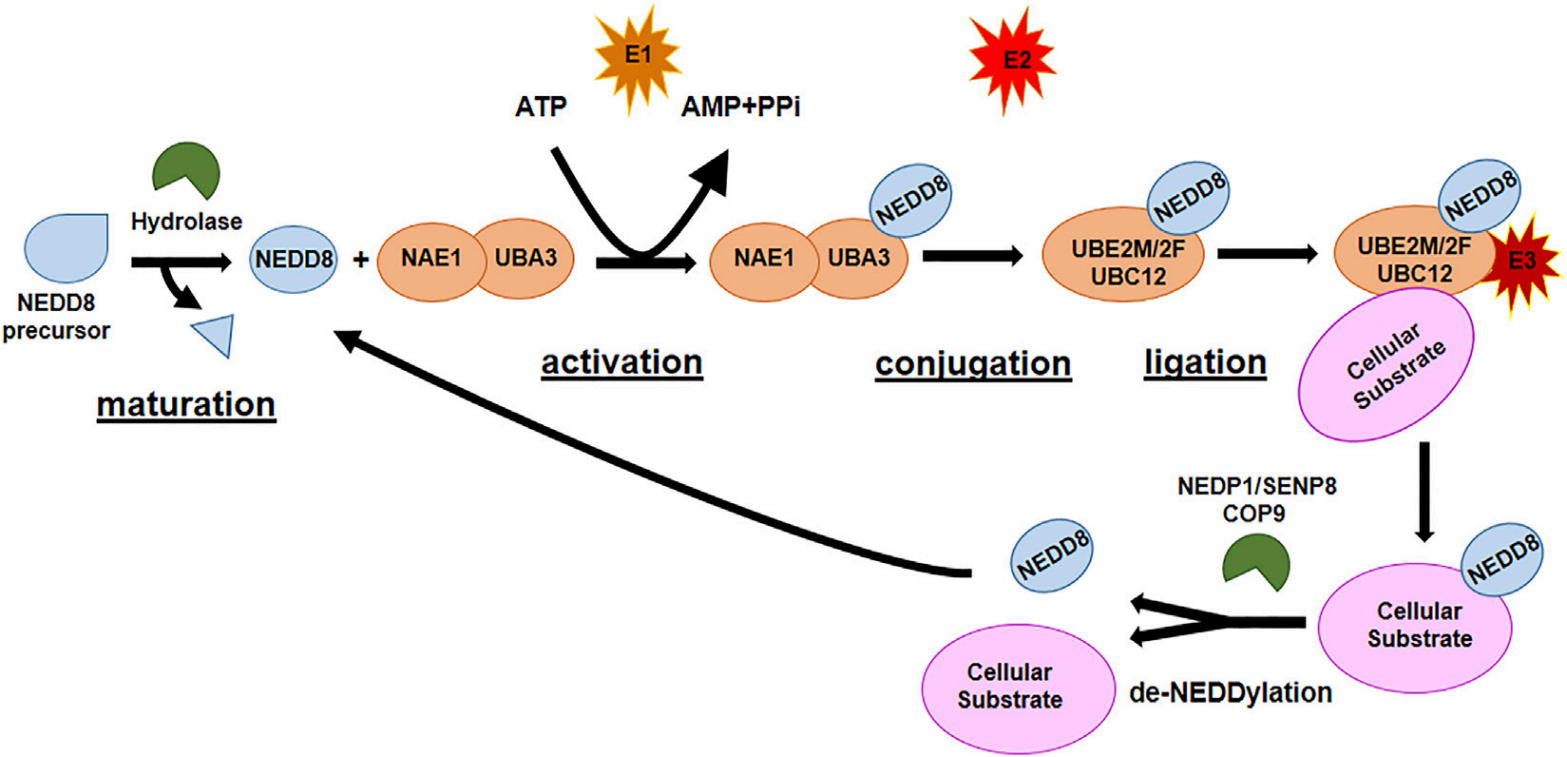
The NEDDylation pathway.

The second most studied Ubl is NEDD8, the protein responsible for NEDDylation. NEDDylation is a post-translational modification consisting of the covalent conjugation of NEDD8 to a Lys residue in the target protein. NEDD8 is the Ubl with the highest homology (80%) with Ub and has analogous activating and conjugating machinery of ubiquitination. As ubiquitination and SUMOylation, NEDDylation is characterized by a sequence of enzymatic cascades involving NEDD8-activating enzyme (NAE) E1, which is a heterodimer of NAE1 and UBA3 and their function is ATP-dependent, NEDD8 ubiquitin-conjugating enzyme (UBE2M, also known as UBC12, and UBE2F) E2, and substrate-specific NEDD8-E3 ligases (Figure 3). The reverse reaction de-NEDDylation, is mediated by several isopeptidases of which only NEDP1/SENP8 and COP9 signalosome are specific for NEDD8, while others are also involved in deubiquitination (Arenas et al., 2021; Zou and Zhang, 2021). The main substrate for NEDDylation are cullins (1–5), a family of proteins core component of cullin–RING E3 ubiquitin ligases (CRLs), responsible of the final step of ubiquitin transfer to a substrate. NEDDylation of cullins is essential for the correct function of the CRL E3 ligases: deregulated NEDDylation leads to dysfunctional ubiquitination and eventually to dysregulation of cell cycle progression and cellular survival. The specific NEDDylation of cullins is referred as canonical NEDDylation and highlight the close connection between NEDDylation and ubiquitination (Vijayasimha and Dolan, 2021; Ilic et al., 2022). However, a growing number of non-cullin targets have also been reported, as p53, Smurf1, PTEN, etc., (Xirodimas et al., 2004; Xie et al., 2014; Xie et al., 2021a), a process referred as non-canonical NEDDylation. Non-canonical NEDDylation serves for the regulation of the activity and function of proteins: it has been reported to affect protein intracellular localization, protein stability both promoting degradation or protecting form it, and protein functions (Vijayasimha and Dolan, 2021; Zou and Zhang, 2021). NEDDylation also regulates physiological and pathological processes such as immunity and inflammatory diseases, cellular response to stress, redox homeostasis, energy metabolism, and tumorigenesis (Arenas et al., 2021; Zou and Zhang, 2021).

## 2 Bone Tumors

Osteosarcoma and chondrosarcoma are the two most common primary solid cancers affecting bone.

Osteosarcoma, the most frequent primary cancer in children and adolescents, has a 5-year survival rate of 70%, and this prognosis drops to 20% in case of metastases at diagnosis. The improvement of systemic chemotherapies, over the past 2 decades, have had a positive impact on the survival of the patients with primary osteosarcoma; however, there are still no effective treatments for patients with recurrences and metastases (Zhao et al., 2021).

Chondrosarcoma is the third most common malignant bone tumor in adults, after multiple myeloma and osteosarcoma, usually occurring in the pelvis or long bones where cancer cells produce hyaline cartilage and form a cartilage matrix (Limaiem et al., 2021). Currently, the standard treatment for chondrosarcoma disease is the surgical resection; however, 10% of patients experiences relapses, and the 5-year survival of patients with high-grade tumors remains below 60%. Furthermore, the management of patients with chondrosarcoma is challenging due to its resistance to conventional chemo and radiotherapy.

## 3 Bone Metastasis

Cancer metastasis represents the final and almost uncontrollable step of cancer progression: tumor cells lose any type of control and spread from the primary to a secondary site. Actually, metastatic spread is an early event that implies several steps starting from cancer cell proliferation and invasion of the stroma, to intravasation, survival in blood vessels, extravasation in the secondary organ, colonization (micrometastatic growth), proliferation/dormancy, and angiogenesis (Ganesh and Massague, 2021).

Bone is frequently interested by metastases, mainly in patients with advanced cancer. Osteolytic and osteoblastic metastasis, the two principal types of bone metastasis, are often accompanied by the so-called skeletal-related events (SREs) characterized by pathological fractures, bone pain, spinal cord compression, and hypercalcemia. These events profoundly affect the quality of life of the patients and reduce overall survival.

Breast cancer (BC) and prostate cancer (PC) are the most prevalent malignancies in women and men, respectively, and often metastasize to bone. PC preferentially develops osteoblastic metastasis, characterized by an enhanced osteoblast activity and the consequent production of osteosclerotic lesions. Bone metastasis from BC, instead, are mainly mixed lesions with both lytic (featured by a predominant osteoclasts activity) and sclerotic components as they appear under radiological investigations.

Despite the advances in cancer treatment, metastases still represent the leading cause of cancer death, and therapeutic advances are desirable to limit their occurrence and, when manifest, to reduce their effects.

## 4 SUMOylation and NEDDylation in Cancer Cell Invasion, Migration, and Metastasis

During tumor progression, tumor cells change their state, acquire the characteristics necessary for local survival, invasion, dissemination, and, eventually, the ability to adapt to unfavorable microenvironments and grow in secondary sites to develop metastases. In this context cancer cells need to adapt to the microenvironment encountered during their spread and cell plasticity is an essential characteristic for metastatic formation. In this scenario, PTMs make an essential contribution by regulating key factors and signaling pathways functional to invasion and metastasis development. In particular, SUMOylation and NEDDylation are potential actors in cancer, especially in processes related to the epithelium-mesenchymal transition (EMT), in the influence exerted on tumor suppressors and oncoproteins, development of metastases and, finally, resistance to therapy.

### 4.1 SUMOylation and NEDDylation in Epithelium-Mesenchymal Transition Process

EMT is considered the driver process of metastasis and often coincides with the acquisition of phenotypic plasticity and stem cell-like properties of the tumor cells. During EMT, cells lose their epithelial features to acquire a mesenchymal phenotype useful for cell migration throughout the supportive connective tissue and, thereafter, to survive into the bloodstream (Wang and Zhou, 2011).

Many transcription factors (TFs) associate with EMT have been identified as targets of SUMOylation (Bogachek et al., 2015). It has been reported that the SUMOylation of hTERT (the catalytic component of the human telomerase enzyme) regulates E-cadherin expression and EMT in BC, thereby promoting migration and invasion of BC cells (Sanyal et al., 2020). In BC, SUMOylation can regulate EMT by acting on the enhancer of zeste homolog 2 (EZH2), a histone-lysine N-methyltransferase, and regulating E-cadherin expression. SUMOylation of E2F1 increases the expression of EZH2 and this leads to E-cadherin repression (Du et al., 2020).

A recent study demonstrates that ZNF451, a SUMO2/3-specific E3 ligase, is a positive regulator of EMT through the SUMOylation of TWIST2 at the K129 residue. SUMOylation stabilizes TWIST2 by inhibiting its ubiquitination and degradation, and, consequently, promotes EMT (Zeng et al., 2021).

Flotillin-1 (Flot-1), a newly discovered SUMOylation target protein positively regulates Snail stability and promotes EMT in metastatic PC (Jang et al., 2019). Flot-1 SUMOylation may serve as a marker to discern malignant PC and, thus, targeting E2 conjugating enzyme Ubc9 could represent a strategy to control EMT in metastatic PC.

ZEB1, the main TF involved in EMT, plays a critical role as a downstream target of many oncogenic pathways, including the PI3K/Akt pathway. By regulating numerous oncoproteins, ZEB1 plays an important role in metastasis. ZEB1 has been reported to be regulated by both SUMOylation and NEDDylation. SUMOylation of ZEB1, as well as its homologue ZEB2, inhibits E-cadherin expression and induce EMT (Long et al., 2005). Silencing of SENP1 decreases ZEB1 protein level, suggesting that de-SUMOylation of ZEB is necessary to activate the TF (Zhang W. et al., 2016). NEDDylation blockade activates PI3K/Akt/mTOR pathway, causes the induction of HIF-1α, and the upregulation of ZEB1 expression (Park et al., 2020). Activation of these pathways causes the induction of tumor cells migration, a hallmark of cancer metastases.

### 4.2 SUMO and NEDD8 Role in Cancer Hypoxic Condition (and Oxygen Sensing)

Intra-tumor hypoxia is a common features in several cancer tissues as the rapid growth of tumor cells is not accompanied by an adequate vasculature development and, consequently, the oxygen availability is reduced (Semenza, 2012). Intra-tumor hypoxia represents an adverse prognostic factor for the patient outcome. Indeed, hypoxia promotes series of modifications in the gene expression that facilitates the adaptation of cancer cells in the hypoxic microenvironment, and therefore, favors invasion and metastasis development.

Hypoxia-inducible factor-1*α* (HIF-1α), a critical mediator in the hypoxic response, is regulated *via* PTMs. Some of these modifications could determine the degradation of HIF-1α through the ubiquitin/proteasome pathway. HIF-1α plays multiple roles in tumor progression and metastasis and the development of inhibitors that target this pathway is of great interest.

SUMO pathway provides control over oxygen sensing, under hypoxic conditions: in hypoxic condition, HIF-1α is SUMOylated and this promotes the degradation of HIF-1α through the recognition of the pVHL E3 ligase complex (Yee Koh et al., 2008). The role of SUMOylation in the cellular adaptation to hypoxia is described by Filippopoulou and colleagues (Filippopoulou et al., 2020). As SUMOylation leads to the inactivation of HIF-1α, SENP enzymes play a key role in the SUMO-dependent regulation of the TF. It has been demonstrated that, during the hypoxic condition in SENP1-deleted embryos and mouse embryonic fibroblast cells, SENP1 is essential for HIF-1α stability (Cheng et al., 2007) and that, in SENP1-overexpressing transgenic mice, HIF-1α level was significantly elevated in nuclei of prostate tissue (Bawa-Khalfe et al., 2010). SENP1 levels increase with PC aggressiveness and correlate with HIF1α levels (Bawa-Khalfe et al., 2010).

NEDD8 directly interacts with HIF-1α and this conjugation enhances the HIF-driven hypoxic signaling; NEDD8 regulates the stabilization of HIF-1α *via* mitochondrial ROS, and this has a great impact on cell survival under hypoxic conditions and oxidative stress (Ryu et al., 2011). Thus, NEDDylation inhibition could be a potential mechanism to counteract tumor growth by targeting the microenvironmental factors.

## 5 SUMOylation and NEDDylation of Tumor-Related Proteins

Many types of cancer show deregulated levels of SUMOylating and NEDDylating enzymes that affect, in different ways, expression and activity of a wide range of oncogenes and tumor suppressor. In this section we will focus on the role of SUMO and NEDD8 in the regulation of tumor suppressors and oncogenes in BC and PC.

### 5.1 Tumor Suppressors

#### 5.1.1 p53

TP53 gene (encoding for p53 protein) is frequently lost, inactivated, or mutated in many types of cancer. In BC, the incidence of p53 mutations differs in different molecular subtypes and mutated p53 is mainly present in Luminal A cancer type (Duffy et al., 2018). Wild type p53 negatively regulates multiple stages of metastasis, while it has been reported that certain p53-mutant proteins, lacking transcriptional activity, drive gain-of-function activities and result in a more aggressive tumor phenotype promoting metastasis. Understanding the mechanisms by which cells loose p53 expression or express gain-of-function mutant p53 will be important to predict tumor behavior and metastatic potential (Powell et al., 2014; Tang et al., 2020).

It has been reported that p53 is SUMOylated by SUMO1 (Chauhan et al., 2021) as well as SUMO2/3, which correlates with a reduction of both activation and repression of a subset of p53-target genes (Stindt et al., 2011). SENP1 abolishes SUMOylation of p53 by direct binding at its p53 DNA-binding domain and, therefore, promotes cancer cell proliferation (Chauhan et al., 2021). Also all the PIAS family members (SUMO E3-ligases) physically interact with p53 and modulate activity and fate of p53 and p53-related family members (Rabellino et al., 2017).

The role of p53 SUMOylation in PC cells is still controversial. It has been reported that androgens affect p53 cytoplasmic localization by inducing the association of G3BP2 (an AR target gene) with RanBP2, a SUMO E3 ligase, thus enhancing p53 SUMOylation: this process associates with PC progression (Ashikari et al., 2017).

On the other hand, p53 SUMOylation, regulated by Mdm2- and ARF-mediated nucleolar targeting, enhances its transcriptional activity, and the consequent acute PTEN inactivation induces growth arrest through the p53-dependent cellular senescence pathway both *in vitro* and *in vivo* (Chen and Chen, 2003; Chen et al., 2005).

The proto-oncogene SKI is overexpressed in a variety of human cancers, and in prostatic cells, SKI negatively regulates p53 by decreasing its level *via* upregulation of Mdm2 activity. This is achieved through SUMOylation by direct interaction of SKI with Ubc9, leading to the enhanced stimulation of Mdm2 SUMOylation and protection from its self-ubiquitination, and consequent degradation, activity (Ding et al., 2012).

p53 activity can also be controlled by NEDDylation. NEDDylation of p53 mediated by the ubiquitin E3 ligase Mdm2 (at K370, K372, and K373) or by FBXO11 (at K320 and K321) leads to inhibition of its transcriptional activity (Xirodimas et al., 2004; Abida et al., 2007).

Also factors upstream or downstream to p53 cascade are regulated by NEDDylation. For instance, NEDDylation of E2F1, a TF playing a pivotal role in cell proliferation and death, modifies the specificity of E2F1 to its target genes and inhibits its pro-apoptotic activity (Aoki et al., 2013). These results suggest that removal of NEDD8 from E2F1 induces apoptosis in response to DNA damage. NEDDylation also has diverse influence on oncogenic signaling pathways such as PI3K/Akt/mTOR and transforming growth factor (TGF)-β (Zuo et al., 2013; Lee et al., 2018a). TGF-β signaling indeed is activated in cancers through the stabilization of the TGF-β type II receptor by NEDDylation (Zuo et al., 2013). The tumor suppressor maspin plays an important role in regulating tumor cell invasion and metastasis. Maspin, highly expressed in normal breast epithelial cells, is downregulated in invasive and metastatic BC cells (Maass et al., 2001). When NEDDylation activity of Mdm2 is inhibited, maspin is induced and that Src-Mdm2-UBC12-NEDD8 axis counteracts the ability of p53 to induce *maspin* (Batuello et al., 2015). This study reveals that in active proliferative cells, in which p53 is elevated yet inactive, NEDD8 could be targeted for therapeutic approaches.

#### 5.1.2 Phosphatase and Tensin Homolog

Phosphatase and tensin homolog (PTEN) is a tumor suppressor often mutated in human cancers (Li et al., 1997). In BC PTEN expression is reduced by 30–40% and, differently from the adjacent normal tissue, NEDDylated PTEN is present in the nuclei of cancer cells (Xie et al., 2021b). Moreover, high level NEDDylation of PTEN in BC patients correlated with poor overall survival, indicating that PTEN NEDDylation might function as a prognostic marker (Xie et al., 2021b). Very recently, it has been reported that high glucose concentrations trigger PTEN NEDDylation and nuclear import and promote tumor development (Du et al., 2021).

Another study reported about the resistance to MLN4924, a selective inhibitor of NAE1 and thus of the NEDDylation modification (Soucy et al., 2009), treatment in cancer patients with complete loss of PTEN, indicating that PTEN is necessary for MLN4924 suppression of tumor growth and that PTEN status is useful to discriminate MLN4924-responsive BC patients (Du et al., 2021).

Once recruited at the plasma membrane, PTEN antagonizes the phosphatidylinositol-3 kinase (PI3K) signaling pathway through de-phosphorylation of phosphatidylinositol 3,4,5-triphosphate (PIP3). PTM’s control PTEN recruitment to plasma membrane, subcellular localization, protein-protein interaction, dual phosphatase activity. Ubiquitination, SUMOylation, and phosphorylation are responsible for PTEN translocation and tumor suppression functions. SUMOylation contributes to influencing nuclear localization and function of PTEN (Bassi et al., 2013). Nuclear PTEN plays pivotal roles in advanced and more aggressive cancers (Whiteman et al., 2002).

SUMOylation acts on the C2 domain of PTEN, at K266, increasing the association with plasma membranes, and at K254, inducing nuclear import (Lee et al., 2018b). It has been reported that SENP1 regulates PTEN stability by blocking SUMO1-dependent ubiquitination and degradation (Bawa-Khalfe et al., 2017).

#### 5.1.3 Retinoblastoma Tumor Suppressor Protein

Retinoblastoma Tumor Suppressor Protein (pRB) is a tumor suppressor with regulatory roles in cell cycle, cell proliferation, and differentiation. pRB inactivation, often encountered in tumor cells, results in uncontrolled growth (Mandigo et al., 2021).

Together with other PTMs, SUMOylation is involved in the regulation of pRB activity during the cell cycle. pRB is SUMOylated during the early G1 phase. This PTM increases its binding with cyclin-dependent kinase (CDK)2 *via* SUMO-SUMO-interaction motif (SIM) interaction, leading to pRB hyper-phosphorylation and E2F-1 TF release, which promotes cell cycle progression (Meng et al., 2016).

SUMO family members mainly interact with hypo-phosphorylated pRB, the activated form of pRB, by SUMOylating the LxCxE binding region in pocket B, a highly conserved region extremely relevant for pRB function, and hence modulating pRB activity. The pRB binding proteins require an intact pocket region for their interaction. Tumorigenic mutations of pRB, within the pocket domain, may lead to a loss of SUMOylation.

SENP1 regulates SUMO1 binding of pRB and Lamin A/C and SUMOylation is required for their optimal interaction. Importantly, this SUMO1-dependent interaction protects pRB and Lamin A/C from proteasome degradation. A dynamic formation of the complex pRB-Lamin A/C depends on SUMOylation in response to changes in cellular conditions. SENP1 regulates pRB SUMOylation levels and indirectly controls pRB ubiquitination and turnover (Sharma and Kuehn, 2016).

### 5.2 Oncoproteins

#### 5.2.1 c-Myc

Deregulation of the oncogenic TF c-Myc (i.e., amplification or overexpression) is frequent in human cancers (Nesbit et al., 1999). In BC, deregulation of c-Myc is found in almost 25% of the patients, and this is often associated with a poor prognosis (Deming et al., 2000; Adler et al., 2006; Chen and Olopade, 2008). c-Myc is often amplified in metastatic PC, and nuclear c-Myc overexpression represents an early event in prostate carcinogenesis (Koh et al., 2010).

c-Myc is dynamically regulated by PTMs, and protein stability and activity are fine-tuned by ubiquitination and SUMOylation and their reverse processes of de-ubiquitination and de-SUMOylation (Chen et al., 2019).

SUMOylation plays a critical role in c-Myc ability to promote oncogenesis, in part by cooperating with c-Myc to maintain the expression of c-Myc target genes involved in mitotic fidelity. Loss of enzymatic activity of SUMO-activating-enzyme (SAE1/2), in particular SAE2, switches a transcriptional subprogram of c-Myc from activated to repressed (Kessler et al., 2012). It has been reported the requirement of SAE2 for Myc-dependent tumor growth and patient survival significantly correlates with SAE1/SAE2 levels in Myc-high tumors (Kessler et al., 2012). These observations reveal a mitotic vulnerability of Myc-driven cancers: the inhibition of SUMOylation impairs Myc-dependent tumorigenesis and might have therapeutic benefits in patients with Myc-driven cancer.

González-Prieto et al. (2015) demonstrated that c-Myc is a target for SUMOylation that determines ubiquitination and proteasomal degradation. A dynamic balance between SUMOylation and ubiquitination is required to properly regulates c-Myc turnover and function in cells, and future studies should address how these PTMs interplay to coordinately regulate c-Myc (Chen et al., 2019).

SENP1 is essential for cancer cell growth and proliferation and its knockdown arrests cell cycle and cell proliferation in different human cancer cell lines. This effect is partially due to the regulation of c-Myc by SENP1: overexpression of c-Myc partially rescued the cell growth inhibition induced by SENP1 knockdown, and SENP1 knockdown inhibits cell proliferation and transformation activity more efficiently in Myc-sensitive BC cell lines than in Myc-insensitive cell lines. Moreover, SENP1 expression is frequently enhanced in human BC cell lines and primary tissues in correlation with the overexpression of c-Myc. Therefore, SENP1-mediated c-Myc de-SUMOylation prevents c-Myc proteasome degradation and enhances c-Myc transcriptional activity, while SENP1 knockdown inhibits cancer cell proliferation through the reduction of c-Myc expression. Thus, by stabilizing c-Myc, SENP1 may contribute to tumorigenesis (Sun et al., 2018).

The COP9 signalosome (CSN) is a protein complex involved in protein degradation, signal transduction, cell cycle, and tumorigenesis. CSN6 enhances cullin-1 NEDDylation and facilitates auto-ubiquitination and degradation of Fbxw7, a Myc E3 ligase; this stabilizes c-Myc, indicating that CSN6 is a positive upstream regulator of c-Myc (Chen et al., 2014). Given that CSN6 is an oncogene often overexpressed in BC and this correlates with Myc protein expression, the inhibition of the CSN6 signaling axis might be therapeutically targeted in Myc-overexpressing cancers.

#### 5.2.2 Protein Inhibitor of Activated STAT

PIAS proteins, initially identified as negative regulators of cytokine signaling by acting mainly on STAT-TFs, act as co-regulators in many pathways (Wnt signaling, p53 pathway, steroid hormone receptor signaling) and regulate a variety of distinct proteins. It is now known that PIAS proteins also act as E3-like ligases by stimulating the association of SUMO with target proteins.

PIAS1 is highly expressed in BC where it regulates tumorigenesis (Liu et al., 2014). Inhibition of the PIAS1 SUMO E3-ligase activity in BC cells promotes bone metastasis in an *in vivo* murine model (Dadakhujaev et al., 2014). Following intra-cardiac injection of BC cells, in which SUMO E3 ligase activity of PIAS1 was disrupted, bone metastasis development was increased. The authors suggested that PIAS1 suppresses BC metastasis through the inhibition of TGF-β signaling, that is thought to promote tumor metastasis *via* the induction of EMT-like behavior in tumor cells at the primary site of tumor formation. These results shed a new light on the interplay between PIAS1 and TGF-β signaling in the regulation of BC bone metastasis (Dadakhujaev et al., 2014).

Nuclear localization of PIAS1 predicts improved BC patient survival (Chanda et al., 2017). PIAS1 triggers SUMOylation of the transcriptional regulator SnoN, which mediates the ability of PIAS1 to suppress the invasive growth of three-dimensional BC cell-derived organoids. The authors suggested that SUMOylated SnoN might also serve as a biomarker in BC and evidence the crucial roles for PIAS1 in BC pathogenesis.

## 6 SUMOylation and NEDDylation in Primary and Metastatic Cancers to Bone

Emerging evidence suggests that tumorigenesis is largely regulated by PTMs and, in particular, SUMOylation and NEDDylation, and their reverse processes, de-SUMOylation and de-NEDDylation, are considered key contributors for cancer initiation and progression. The contribution of these PTMs to neoplasia is related to the types of cancer and the specific context. Below we give an overview of the literature on the roles of SUMOylation and NEDDylation on bone malignancies focusing on osteosarcoma, chondrosarcoma and breast and prostate cancers, which often metastasize to bone.

### 6.1 SUMOylation and NEDDylation in Osteosarcoma

Wang and colleagues elucidated a potential role of SENP1 in osteosarcoma development and invasiveness by the observation that a positive feedback loop exists between HIF-1α and SENP1 (Wang et al., 2018). The involvement of SENP1/HIF-1α axis in the regulation of cell viability, EMT and invasion under hypoxia shed a light on its role as potential therapeutic target for osteosarcoma treatment (Wang et al., 2018).

SENP2, which has a de-SUMOylating activity, is downregulated in both osteosarcoma tissues and MG63 osteosarcoma cell line. SENP2 regulates the proliferation, migration, and invasion of osteosarcoma cells *via* the degradation of SOX9, and in this way, it exerts its tumor suppressor function (Pei et al., 2018).

Osteosarcoma cells and tissues exhibit SENP5 overexpression, important for cell division and maintenance of mitochondrial morphology and function. SENP5 controls osteosarcoma cell proliferation by regulating G2/M arrest and apoptosis, and therefore could represent a reliable strategy for osteosarcoma treatments (Wang and Zhang, 2014).

Ubc9 is overexpressed in both osteosarcoma tissues and cell lines. Ubc9 silencing in U-2OS cells decreases their ability to proliferate, migrate, and form colonies while weakly increases the apoptotic rate. Moreover, Ubc9 silencing improves the sensitivity of U-2OS to HSV-TK/GCV chemotherapy in both *in vitro* and *in vivo* systems (Zhang et al., 2018). A new strategy has been proposed to improve sensitivity of osteosarcoma cells to chemotherapy by inducing de-SUMOylation of connexin 43 (Cx43), a component of gap junctions, and increasing free Cx43 levels, important for recovery of normal cellular functions (Zhang et al., 2018).

Talin, a key component of focal adhesions (FAs), plays an important role in the regulation of cell migration, protein-protein interaction, and cell signaling, and is regulated by SUMOylation in U-2OS cells. Talin SUMOylation affects both the formation and function of FAs and is therefore important for tumor cell migration. Additionally, inhibition of SUMOylation reduces the proliferation rate of cancer cells mediated by Talin (Huang et al., 2018).

In human osteosarcoma, it has been demonstrated that MLN4924 (Pevonedistat) reduces cell viability, induces DNA damage, senescence, and apoptosis. Indeed, MLN4924 inhibits NEDDylation of cullins but, at the same time, induces the accumulation of several pro-survival oncogenic proteins, such as cyclin E and Mcl-1, and thus the anticancer properties of MLN4924 are reduced (Zhang Y. et al., 2016). The same authors demonstrated that MLN4924 alone has only a slight apoptotic effect in osteosarcoma cells while the concomitant inhibition of Mcl-1, an anti-apoptotic protein, considerably enhances the MLN4924 action, suggesting that combining treatments is important to achieve better results in anticancer therapy in osteosarcoma (Zhang et al., 2017).

### 6.2 SUMOylation and NEDDylation in Chondrosarcoma

In chondrosarcoma, it has been shown that the increased expression of SUMO1 and SUMO2/3 is associated with tumor progression and aggressiveness; SUMO2/3 levels are also correlated with poorer survival. De-differentiated chondrosarcoma cell lines, compared to non-malignant cells, are particularly sensitive to SUMO E1 inhibitor ML792, which reduces cell proliferation and viability in a dose-dependent manner. The authors speculated on the possibility of using the SUMO E1 inhibitor, *in vivo*, for the treatment of aggressive chondrosarcoma (Kroonen et al., 2021).

It has been reported that MLN4924 targets NAE-activated NEDDylation and suppresses the growth of chondrosarcoma cells by inhibiting the phosphorylation of histone H3 and G2/M cell cycle arrest. MLN4924 in human chondrosarcoma cells induces caspase activation, cellular stress, and apoptosis and it has been proposed that the inhibition of the NEDDylation pathway could represent a novel potential strategy for the treatment of chondrosarcoma (Wu et al., 2018).

### 6.3 SUMOylation and NEDDylation in Prostate Cancer

Steroid receptors, such as androgen receptors (ARs), are targets of SUMOylation. Increased ARs expression drives PC development through the upregulation of downstream targets and co-factors in cell proliferation. ARs are SUMOylated at the K386 and K520 residues (Poukka et al., 2000) and SUMOylation represents a modulating system able to restrict steroid receptor activity; a rapid and reversible mechanism to attenuate AR function.

Some authors have suggested that SENP1 expression might contribute to the malignant progression of human PC (Li et al., 2013). SENP1 is overexpressed in prostatic intraepithelial neoplasia (PIN), a pre-cancerous prostatic lesion, and in PC tissue with respect to the normal ones. It has been demonstrated that the increased expression of SENP1 in PIN and PC is induced by androgens and IL-6, and that SENP1 induction enhances the transcriptional activity of ARs and c-Jun and modulates cyclin D1 expression (Cheng et al., 2006). In transgenic mice, prostate gland-specific overexpression of SENP1 is related to early PIN development, suggesting that SENP1 could be responsible for the initiation and promotion of PC (Cheng et al., 2006).

SENP1 might take part in the pathogeneses of PC by activating the HIF1α signaling pathway and its downstream target gene, vascular endothelial growth factor (VEGF) (Bawa-Khalfe et al., 2010; Wang et al., 2019); moreover, SENP1 expression directly correlates with PC aggressiveness and recurrence, and SENP1 silencing in highly metastatic PC cells counteracts the ability to metastasize to the bone (Wang et al., 2013). SENP1-mediated regulation of MMP2 and MMP9 is partially responsible for the triggering of bone metastasis. The same authors suggested that SENP1 could be used as a prognostic marker and as a therapeutic target for metastasis in PC patients (Wang et al., 2013).

Huang et al. (2012), analyzed the effect of an active compound, extracted from Chinese medicinal herb, the Triptolide, on PC. Triptolide shows anti-tumor activity on PC that involves downregulation of SENP1, restoration of SUMOylation/de-SUMOylation balance, and negative regulation of ARs and c-Jun expression (Huang et al., 2012).

SUMO1, Uba2, and Ubc9 upregulation in PCs could lead to enhanced SUMOylation of the TF ETS variant 1 (ETV1), and blocking ETV1 tumor-promoting activity by SUMOylation could suppress the growth of PC (Oh et al., 2021).

In PC, SENP3 promotes VEGF expression and facilitates the development of new blood vessels, contributing to cancer cell survival under hypoxic conditions (Huang et al., 2009). Therefore, by regulating angiogenesis, SUMO plays an additional role in the cellular adaptation to hypoxia and favors PC progression.

Collectively, SENP1 and/or SENP3 show carcinogenic properties by promoting AR-dependent and AR-independent cell proliferation, activating the HIF-1α signaling pathway, increasing VEGF expression, and thus supporting angiogenesis, in PC.

An interesting study identified a new signaling pathway in the regulation of cancer metastasis: SUMOylation of reptin, a component of ATP-dependent chromatin remodeling complexes that function as antagonistic regulators of the Wnt/β-catenin pathway, modulates the metastasis suppressor gene *KAI1* also in PC cells with metastatic behavior, favoring cells invasiveness (Kim et al., 2006). This represents a novel link between SUMO modification and cancer metastasis (Baek, 2006).

It has been demonstrated that targeting the NEDDylation pathway by MLN4924 suppresses the growth of both hormone-sensitive (LNCap) and hormone-resistant (DU145) PC cells and this can be considered as an attractive anticancer strategy (Wang et al., 2014).

### 6.4 SUMOylation and NEDDylation in Breast Cancer

Claudin 6 (CLDN6) appears to be able to regulate the HIF-1α pathway through SENP1 in BC cells and BC patients with primary CLDN6 loss are more prone to have tumor metastasis, due to a lack of feedback mechanism that impairs HIF-1α stability. The authors demonstrated that CLDN6 is transcriptionally upregulated by HIF-1α, and in turn, reduces HIF-1α stability counteracting its SENP1-mediated de-SUMOylation. They demonstrated that CLDN6 attenuates hypoxia-induced tumor metastasis *via* SUMOylation and they speculated that these findings might provide a novel strategy to treat BC and a mechanism to be exploited in anti-cancer therapy (Jia et al., 2020).

Loss of SENP3 favors macrophages polarization towards M2 (CD206^+^) in BC microenvironment and results in increased proliferation and migration of BC cells. SENP3 downregulation depends on the hyper-SUMOylation of Akt1 followed by its hyper-phosphorylation and activation which is responsible for M2 polarization in the tumor microenvironment and for tumor progression (Xiao et al., 2021).

Lorente and colleagues analyzed the role of SUMO1 pathway in controlling BC tumorigenesis, cancer survival and invasiveness by either depleting SUMO1 and Ubc9 or by exposing to ginkgolic acid C15:1 or 2-D08 (two SUMOylation inhibitors) (Lorente et al., 2019). Inhibition of protein SUMOylation induces autophagic-mediated cancer cell death through the upregulation of tribbles pseudokinase 3 (TRIB3) and inhibits cancer cell invasiveness through the activation of the small GTPase rac1 (Lorente et al., 2019).

In basal-like BC, characterized by an aggressive clinical outcome and metastasis, the cleavage of syndecan-1, a membrane-bound proteoglycan, *via* Akt SUMOylation, promotes cells proliferation and cancer progression (Nadanaka et al., 2021).

E1 enzymes, SAE1 and SAE2, measurements represent a feasible tool to predict the potential malignancy of BC. SUMO E1 enzyme is involved in Myc-driven tumors; SAE2 is required in supporting Myc-dependent carcinogenesis, and its depletion, that triggers mitotic defects, reduces Myc-dependent BC (Kessler et al., 2012).

SUMO E2 Ubc9 is frequently upregulated in neoplastic tissues. In BC hormone receptor-positive subtypes there has been detected higher levels of Ubc9 with respect to hormone receptor-negative and invasive BC lesions, and in metastatic BC, Ubc9 expression is decreased in comparison to the corresponding normal breast or primary BC (Moschos et al., 2010).

PIAS3, through the SUMOylation of the ubiquitin E3 ligase Smurf2, suppresses the invasiveness of BC cell-derived organoids; these data are important and deserve to be investigated as the SUMOylation of PIAS3-Smurf2 could suppress the invasion and metastasis of BC.

Licciardello and colleagues reported a selective interaction between the NOTCH1 signaling pathway and the SUMOylation cascade. In BC, upon inhibition of SUMOylation, NOTCH1-activated cells proceed slower through the cell cycle before entering apoptosis. Moreover, NOTCH1 activation depletes unconjugated SUMO and increases sensitivity to the inhibition of SUMOylation cascade (Licciardello et al., 2015).

MKK7, a JNK-specific MAP2K, undergoes NEDDylation in human BC cells. MKK7 NEDDylation is affected by different environmental factors; indeed, MKK7 NEDDylation is induced in response to stressful stimuli (nutrient starvation, free fatty acids, and sorbitol osmotic stimulation). These scholars sustain that MKK7 NEDDylation could help BC cells to survive during these stresses by limiting JNK activation (Zhu et al., 2016).

It has been reported that UBC12-mediated sterol regulatory element-binding protein 1 (SREBP-1) NEDDylation is enhanced in BC and contribute to BC growth, aggressiveness, to worsen metastatic tumor prognosis and to compromise overall patient survival rates (Heo et al., 2020).

Talin can be subjected to post-translational modification by SUMOylation in BC cells (MDA-MB231). SUMOylation inhibition increased number, size, and turnover-time of FAs in cancer cells, potentially identifying in SUMOylation a novel mechanism underlying cell migration (Huang et al., 2018).

SREBP-1 is a NEDDylation substrate for UBC12, a NEDD8-conjugating enzyme: NEDDylation stabilizes SREBP-1 and decreases its ubiquitination. It has been demonstrated that SREBP-1 and UBC12 levels raise in BC patients, and a higher SREBP-1 expression have been identified in patients with metastasis (Heo et al., 2020). This is in agreement with the finding that SREBP1 overexpression promotes EMT, cancer progression, and metastasis in BC cells (Zhang et al., 2019).

**FIGURE 4:**
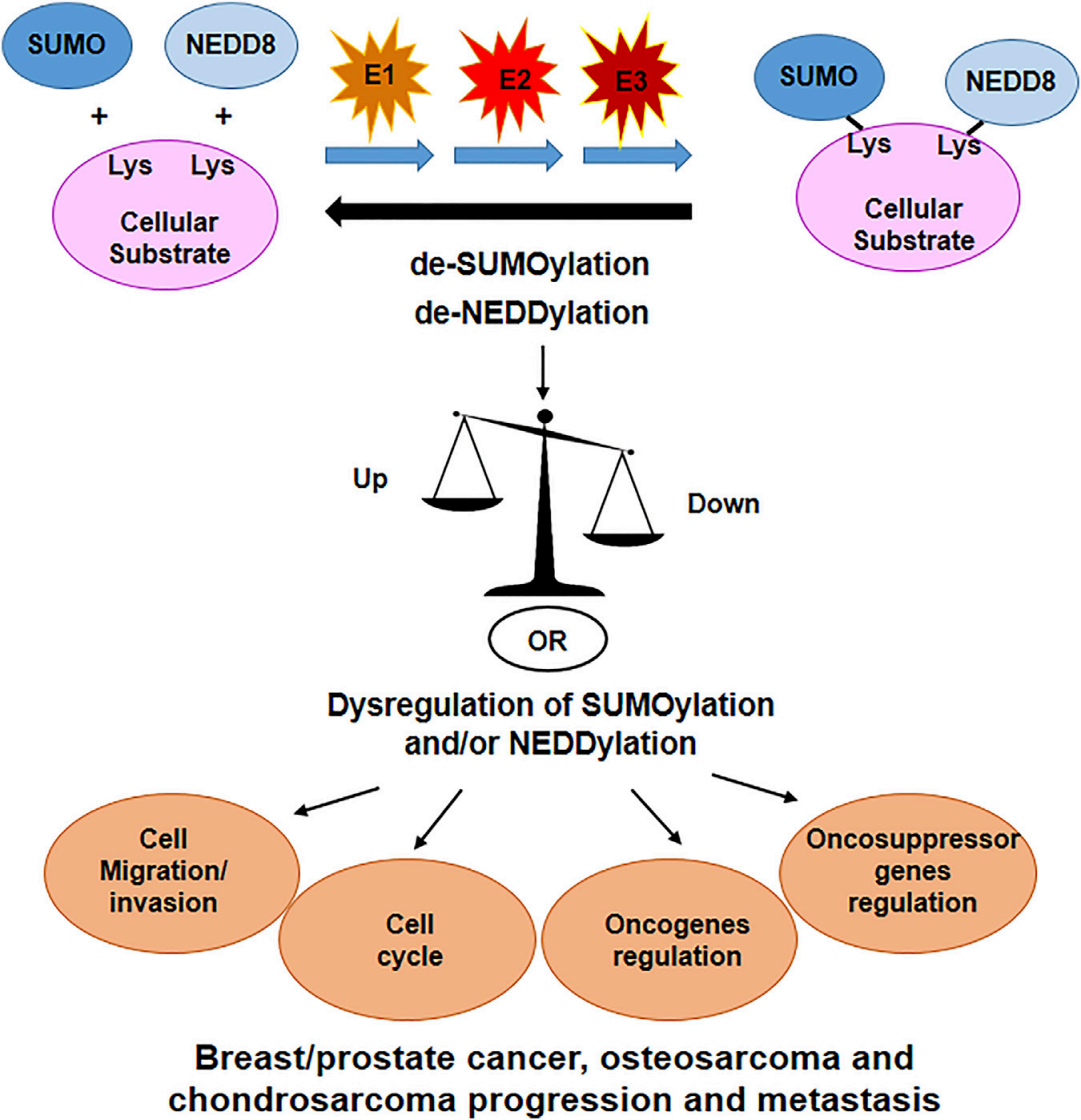
SUMOylation and NEDDylation are important PTM’s that regulates many cellular proteins. Dysregulation of these processes have been linked to alteration in cell cycle, cell migration and invasion, oncogenes and oncosuppressor genes regulation, altogether contributing in the progression of tumors and eventually metastasis formation.

The contribution of SUMOylation and NEDDylation in the modulation of the cellular processes involved in cancer progression and metastasis is summarized in Figure 4.

## 7 SUMOylation and NEDDylation in Cancer Drug Resistance

One of the prevailing options for combating aggressive tumors is the use of chemotherapeutic agents; however, some cancers often develop drug resistance leading to treatment ineffectiveness, relapse, and ultimately a poor prognosis. Therefore, it is urgent to understand the reason for drug resistance and to develop a new strategy to effectively counter it with a rational therapy.

In BC cells, it has been reported that SENP2 reduced doxorubicin resistance, at least partially, by suppressing the NF-κB pathway. SENP2 de-SUMOylates NF-κB essential modulator (NEMO), the principal modulator of NF-κB, and consequently suppresses NF-κB activity and cell survival (Gao et al., 2019). This novel function of SENP2 should be therefore important to sensitize BC cells to doxorubicin.

In addition, inactivation of NEDDylation in ER-positive BC cells stimulates the sensitivity to Fulvestrant and this could be considered in combination with endocrine therapy to develop synergistic effect on ER-positive BC.

## 8 NEDDylation and SUMOylation Inhibitors

To date, there is an urgent need to identify novel therapeutic targets to treat, with minimal side effects, bone diseases. Among these, a crescent number of SUMOylation and NEDDylation inhibitors, are emerging as attractive targets for cancer therapy.

### 8.1 MLN4924 (Inhibitor of NEDD8-Activating Enzyme)

Inhibition of protein NEDDylation has been recently proposed as an attractive anticancer strategy. MLN4924 is a potent and highly selective inhibitor of NAE.

By inactivating the first phase of the NEDDylation cascade, MLN4924 leads to the suppression of BC cells’ stemness and their sensitization to tamoxifen, both in *in vitro* and *in vivo* cancer models (Soucy et al., 2009; Yin et al., 2019).

However, in p53-null BC cell lines, NEDDylation blockade increases cell migration by activating the PI3K/Akt/mTOR/Slug signaling axis. Conversely, in cancer cell lines expressing wild-type p53, NEDDylation blockade induces p53 transcriptional activity and promotes proteasome degradation of Slug, which results in reduced or stable cell migration. Therefore, careful attention should be paid to p53 status before using MLN4924 or NEDDylation-targeted anticancer drugs (Kim et al., 2021). The disruption of p53, in combination with downregulation of NAE1, could be potential prognostic factors that accelerate cancer metastasis.

Park et al. (2020) suggested that attention should be paid to the use of MLN4924 as an anticancer drug; in fact, NEDDylation blockade induces HIF-1α *via* activation of PI3K/Akt/mTOR pathway and, thereby, induces ZEB1 expression that is linked to poor patient survival and active metastasis.

Estrogen-related receptor (ERR) β is normally downregulated in BC cells and, when overexpressed in BC patients, it positively correlates with improved prognosis and prolonged relapse-free survival. MLN4924 can restore the ERRβ expression and, concomitantly, reduces cell proliferation and migration of BC cells, indicating that inhibition of NEDDylation can be approached as a novel and effective strategy to treat BC (Naik et al., 2020).

NEDDylation inhibition by MLN4924 exerts anticancer effect not only by inducing cell apoptosis, senescence, and autophagy, but also by influencing the functions of tumor microenvironment components (cancer-associated fibroblasts, cancer-associated endothelial cells, macrophages, etc.), that play important roles in tumorigenesis (Zhou et al., 2019).

Finally, a therapy combining MLN4924 and radiation or chemotherapeutics may represent an alternative to counteract cancer development and spread. MLN4924 has been shown to sensitize breast and pancreas cells to ionizing radiation (Wei et al., 2012; Yang et al., 2012).

### 8.2 ML-792 (SUMO-Activating Enzyme Inhibitor) and its Derivative

ML-792, a selective and potent blocker of SAE activity and SUMOylation, acts by inhibiting cancer cells proliferation. This effect of ML-792 on cell viability was enhanced in cells with MYC oncogene amplification, suggesting its potential therapeutic application against MYC-amplified tumors (He et al., 2017).

ML-93, a derivative of ML-792, has been recently studied in pancreatic ductal carcinoma. At the nanomolar range, ML-93 suppresses both human pancreatic cancer cell line growth and tumor growth, in an *in vivo* pancreatic ductal carcinoma xenograft model (Biederstadt et al., 2020). In particular, in an aggressive pancreatic ductal adenocarcinoma subgroup, characterized by the hyperactivation of MYC pathways and poor prognosis due to the lacking of clear treatment options, pharmacological SUMO inhibition could represent an interesting approach.

TAK-981, another small molecule derived from ML-792, is a SUMO E1 inhibitor that acts as a SAE inhibitor by forming a SUMO-TAK-981 adduct, within the enzyme catalytic site. To date, TAK-981 is the only SUMO inhibitor tested in ongoing clinical trials in humans (Langston et al., 2021). Most of the studies analyzed the effect of TAK-981 in combination with different drugs (i.e., anti-CD38 antibodies Mezagitamab and Daratumumab; immunotherapy agents Pembrolizumab and Avelumab; anti-CD20 antibody; Rituximab; anti-EGFR antibody Cetuximab) in different types of cancers and metastatic solid tumors (clinical trials: NCT04381650, NCT04065555, NCT03648372, NCT04074330, and NCT04776018).

These are ongoing studies, and the results have not yet been identified.

### 8.3 Ginkgolic Acid

Ginkgolic acid, a group of alkyl phenols derived from Ginkgo *biloba*, is one of the first natural products discovered that inhibits the formation of SUMO-E1 complex in the SUMOylation process (Fukuda et al., 2009). Ginkgolic acid shows anticancer activity and inhibits cancer cell migration. In BC cells, ginkgolic acid exerts these properties through the inhibition of NEMO SUMOylation, inhibition of IκBα degradation, and eventually NF-κB activity impairment. These processes lead to downregulation of metastasis-related genes, as uPA, PAI-1, CXCR4, and MMP-9, and control metastatic dissemination of BC cells (Hamdoun and Efferth, 2017). The treatment of BC cells with ginkgolic acid (C15:1) determines the blockade of the SUMO1 pathway and subsequent cell invasion through RAC1 SUMOylation inhibition. This process diminishes the amount of active RAC1 for cell migration and invasion (Lorente et al., 2019). While potentially interesting, ginkgolic acid is not highly specific in inducing the inhibition of the SUMO pathway only (it also exhibits antidepressant, antifungal, and antimicrobial activities), and its low bioavailability must be evaluated before its clinical use (Brackett et al., 2020).

## 9 Conclusion

SUMOylation and NEDDylation, as post-translational modifiers, act on cancer cells and/or control proteins involved in cancer progression and metastasis. These PTMs, therefore, represent promising/attractive therapeutic targets to counteract tumor development and to enhance antitumor immunity. Understanding how these processes affect the signaling pathways involved in cancer development and progression could provide useful information about the development of specific drugs and therapeutic strategies tailored to individual patients.

Different points of crosstalk may occur between SUMOylation and NEDDylation, including competition for common amino acid residues or regulation of the NEDD8 conjugation machinery by SUMO. The interaction between SUMOylation and NEDDylation should be considered when a therapeutic program is designed to avoid ineffectiveness.

Metastasis is the leading cause of BC and PC treatment failure. The new light shed on the relationship between PTMs and cancer progression and/or metastasis development is important to consider. Further studies are needed to better characterize and target these novel PTMs with the goal to counteract the metastatic spread.
